# Neuroinflammation generated by HIV-infected microglia promotes dysfunction and death of neurons in human brain organoids

**DOI:** 10.1093/pnasnexus/pgae179

**Published:** 2024-04-29

**Authors:** Weili Kong, Julie Frouard, Guorui Xie, Michael J Corley, Ekram Helmy, Gang Zhang, Roland Schwarzer, Mauricio Montano, Peter Sohn, Nadia R Roan, Lishomwa C Ndhlovu, Li Gan, Warner C Greene

**Affiliations:** Michael Hulton Center for HIV Cure Research at Gladstone, San Francisco, CA 94158, USA; Gladstone Institute of Virology, San Francisco, CA 94158, USA; Michael Hulton Center for HIV Cure Research at Gladstone, San Francisco, CA 94158, USA; Gladstone Institute of Virology, San Francisco, CA 94158, USA; Department of Urology, University of California, San Francisco, San Francisco, CA 94143, USA; Michael Hulton Center for HIV Cure Research at Gladstone, San Francisco, CA 94158, USA; Gladstone Institute of Virology, San Francisco, CA 94158, USA; Department of Urology, University of California, San Francisco, San Francisco, CA 94143, USA; Division of Infectious Diseases, Department of Medicine, Weill Cornell Medicine, New York, NY 10021, USA; Brain and Mind Research Institute, Weill Cornell Medicine, New York, NY 10021, USA; Michael Hulton Center for HIV Cure Research at Gladstone, San Francisco, CA 94158, USA; Gladstone Institute of Virology, San Francisco, CA 94158, USA; Michael Hulton Center for HIV Cure Research at Gladstone, San Francisco, CA 94158, USA; Gladstone Institute of Virology, San Francisco, CA 94158, USA; Michael Hulton Center for HIV Cure Research at Gladstone, San Francisco, CA 94158, USA; Gladstone Institute of Virology, San Francisco, CA 94158, USA; Michael Hulton Center for HIV Cure Research at Gladstone, San Francisco, CA 94158, USA; Gladstone Institute of Virology, San Francisco, CA 94158, USA; Gladstone Institute of Neurological Disease, San Francisco, CA 94158, USA; Michael Hulton Center for HIV Cure Research at Gladstone, San Francisco, CA 94158, USA; Gladstone Institute of Virology, San Francisco, CA 94158, USA; Department of Urology, University of California, San Francisco, San Francisco, CA 94143, USA; Division of Infectious Diseases, Department of Medicine, Weill Cornell Medicine, New York, NY 10021, USA; Brain and Mind Research Institute, Weill Cornell Medicine, New York, NY 10021, USA; Brain and Mind Research Institute, Weill Cornell Medicine, New York, NY 10021, USA; Helen and Robert Appel Alzheimer's Disease Research Institute, Weill Cornell Medicine, New York, NY 10021, USA; Michael Hulton Center for HIV Cure Research at Gladstone, San Francisco, CA 94158, USA; Gladstone Institute of Virology, San Francisco, CA 94158, USA; Departments of Medicine and Microbiology and Immunology, University of California, San Francisco, San Francisco, CA 94143, USA

**Keywords:** HAND, microglia, inflammation, neuronal dysfunction, human iPSC-derived brain organoids

## Abstract

Despite the success of combination antiretroviral therapy (ART) for individuals living with HIV, mild forms of HIV-associated neurocognitive disorder (HAND) continue to occur. Brain microglia form the principal target for HIV infection in the brain. It remains unknown how infection of these cells leads to neuroinflammation, neuronal dysfunction, and/or death observed in HAND. Utilizing two different inducible pluripotent stem cell-derived brain organoid models (cerebral and choroid plexus [ChP] organoids) containing microglia, we investigated the pathogenic changes associated with HIV infection. Infection of microglia was associated with a sharp increase in CCL2 and CXCL10 chemokine gene expression and the activation of many type I interferon stimulated genes (MX1, ISG15, ISG20, IFI27, IFITM3 and others). Production of the proinflammatory chemokines persisted at low levels after treatment of the cell cultures with ART, consistent with the persistence of mild HAND following clinical introduction of ART. Expression of multiple members of the S100 family of inflammatory genes sharply increased following HIV infection of microglia measured by single-cell RNA-seq. However, S100 gene expression was not limited to microglia but was also detected more broadly in uninfected stromal cells, mature and immature ChP cells, neural progenitor cells and importantly in bystander neurons suggesting propagation of the inflammatory response to bystander cells. Neurotransmitter transporter expression declined in uninfected neurons, accompanied by increased expression of genes promoting cellular senescence and cell death. Together, these studies underscore how an inflammatory response generated in HIV-infected microglia is propagated to multiple uninfected bystander cells ultimately resulting in the dysfunction and death of bystander neurons.

**Significance Statement**

Even after the introduction of effective antiretroviral therapy (ART), mild forms of HIV-associated neurocognitive disorder (HAND) are found in 30–50% of HIV-positive people. To dissect the pathogenic mechanisms underlying development of HAND, two inducible pluripotent stem cell-derived human brain organoids were used as experimental models. Our results are consistent with an initial inflammatory response in infected microglia leading to the activation of type I IFN-regulated genes and proinflammatory genes. This inflammatory response subsequently spreads to other types of uninfected brain cells including neurons, generating a toxic miocroenvironment that disrupts normal neuron function and survival. This response is blunted but not eliminated by the introduction of ART, consistent with the persistence of HAND in the post-ART era.

## Introduction

Approximately 39 million people worldwide are infected with HIV, with 1–2 million new infections occurring each year. Despite increasingly widespread use of highly effective antiretroviral therapy (ART), mild cases of HIV-associated neurocognitive disorder (HAND) continue to occur in 30–50% of patients living with HIV (1–3). Affected individuals present with a broad spectrum of neurological deficits including decreased attention, focus, and memory; lack of motivation; irritability; depression; and slower physical movements. All of these symptoms can negatively impact quality of life.

HIV infection of the brain usually occurs within 2–4 weeks after initial infection, HIV invades the central nervous system (CNS) (4). T cells and monocytes infected with the virus are capable of crossing the blood–brain barrier (5). In turn, HIV is transmitted to CNS-resident tissue macrophages and microglia (6–8) that comprise between 0.5 and 16% of all cells in the adult brain (9, 10). These target cells, especially microglia, likely play a prominent role in HAND pathogenesis (11–14).

HIV infection of microglia alters their state of activation, viability, and metabolism (15–18). Within infected microglia, expression of HIV gp120 Env, Vpr, Tat, and viral RNA are readily detectable. In addition, proinflammatory cellular pathways leading to the production of interleukin-1 (IL-1), interleukin-6 (IL-6), tumor necrosis factor alpha (TNF-α), and reactive oxygen species are activated and could play a role in neuronal injury and apoptosis (18–26). Of note, most HAND research so far involves cell culture systems where viral proteins are overexpressed or transgenic models (27–30), that fail to fully recapitulate human immune responses. While HIV infection of primary human fetal microglia has been documented (31), these cells are not widely accessible, limiting their utility for molecular studies of HIV-induced pathologies underlying HAND pathogenesis.

Preclinical studies of HAND have extended to the use of various in vivo models including not only transgenic and humanized mice and rats but also nonhuman primate models (27–30). However, the intrinsic cost and lack of broad availability of these models have limited their utility. We have elected to use inducible pluripotent stem cells (iPSC's) to generate human brain organoids that are comprised of a mixture of different types of brain cells including microglia. These brain organoids recapitulate the diverse cellular composition and three-dimensional (3D) environment found in the fetal human brain. Following HIV infection of two different organoid models (cerebral organoids and choroid plexus organoids), we have identified the cells are targeted by the virus and defined the molecular events that are evoked by infection using a variety of techniques including single-cell RNA-seq.

## Results

### HIV principally infects microglia in cerebral brain organoids but changes in the microenvironment promote death of bystander neurons

To generate the cerebral organoids containing microglia cells, we mixed wild-type (WTC11) iPSCs with a reprogrammed line, MG-iPSC, where doxycycline drives expression of different transcription factors needed for differentiation of microglia (32). After culturing the organoids for 5 weeks, we measured expression of cell markers identifying microglia, neurons, astrocytes, and neuronal progenitor cells (Fig. S1). All four cell types were present, and the cerebral organoids exhibited a predominantly dorsal forebrain region specification. Ventricle-like structures filled with fluid and lined by Sox2/Nestin-positive neural progenitors were observed and found. Additionally, beta-tubulin III (Tuj1)/MAP2-positive neurons were detected in the outer layer (Fig. S1B). Notably, astrocytes were only rarely observed at early time points but appeared after 50 days (Fig. S1C). IBA1-positive microglia were readily detected, often surrounded by intercalating neurons (Fig. S1D).

**Fig. 1.**
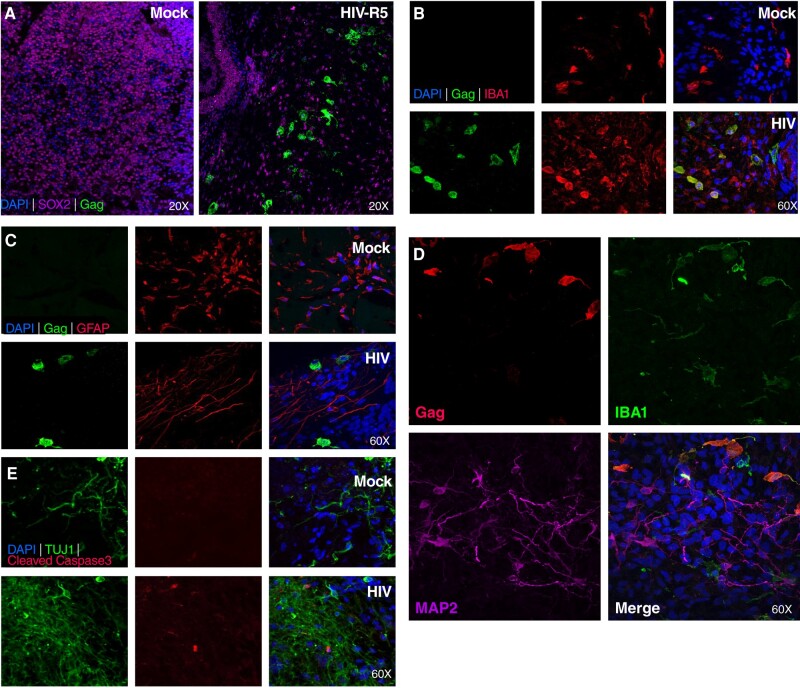
HIV infects microglia in cerebral organoids. A) Cerebral organoids were either mock-infected or infected with R5-tropic HIV-1 ADA on day 14 and analyzed on day 54 of culture. Anti-Gag immunofluorescence staining was employed to detect HIV-infected cells while anti-SOX2 immunostaining was used to identify neuronal progenitor cells. DAPI was used to stain DNA of all cells. Magnification is indicated for each panel. B) Confocal images of antibody immunostaining for IBA1 and HIV Gag in mock-infected (upper row) and HIV-infected organoids (lower row). Note co-immunostaining of a subset of IBA1-positive microglia, and Gag positive HIV-infected cells. Magnification is 60× for all panels. C) Confocal images of immunostaining for astrocytes (GFAP) and HIV Gag. Note an absence of detectable co-immunostaining. Magnification is 60× for all panels. D) Cerebral organoids were infected with HIV-1 ADA on day 14 and analyzed on day 54. Immunofluorescence staining detecting HIV (anti-Gag immunostaining), neuronal cells (anti-MAP2 immunostaining), and microglia cells (anti-IBA1 immunostaining). Magnification is 60× for all panels. E) Immunofluorescent confocal images of neurons stained with anti-TUJ1 antibody, antibody and cleaved (activated) Caspase-3, and nuclear DNA staining with DAPI. Magnification is 60× for all panels.

To ensure a high HIV infection rate in cerebral organoids, we initiated infection at day 14 representing an early stage of organoid development. Cultures were continued for an additional 40 days post-infection (dpi), followed by microscopic examination and immunostaining. HIV infection was detected using anti-Gag antibody (Fig. 1). As expected, no Gag staining was detected in mock-infected organoids. Infected cells exhibited the morphology of microglia (Fig. 1A), and their cellular classification was confirmed by dual staining with IBA1, a microglia marker (Fig. 1B) and anti-Gag to identify infected cells. To determine whether HIV also establishes productive infection in astrocytes, the most abundant immune cells in the CNS, cerebral brain organoids were infected with HIV-1 and analyzed approximately 60 days later. No productively infected astrocytes were identified (Fig. 1C). Of note, while neurons were not directly infected (Fig. 1D), progressive death of these cells was observed in infected but not uninfected organoids (Fig. 1E).

To investigate the effects of ART on HIV infection, 3-month-old cerebral organoids were infected with ADA for 3 days, then subjected to ART treatment. Supernatants were collected every 3 days and measured for p24 by ELISA. ART treatment significantly reduced levels of p24-Gag measured by ELISA, a finding confirmed by anti-p24-Gag immunostaining (Fig. S2A and B).

### HIV infects microglia in ChP brain organoids

**Fig. 2.**
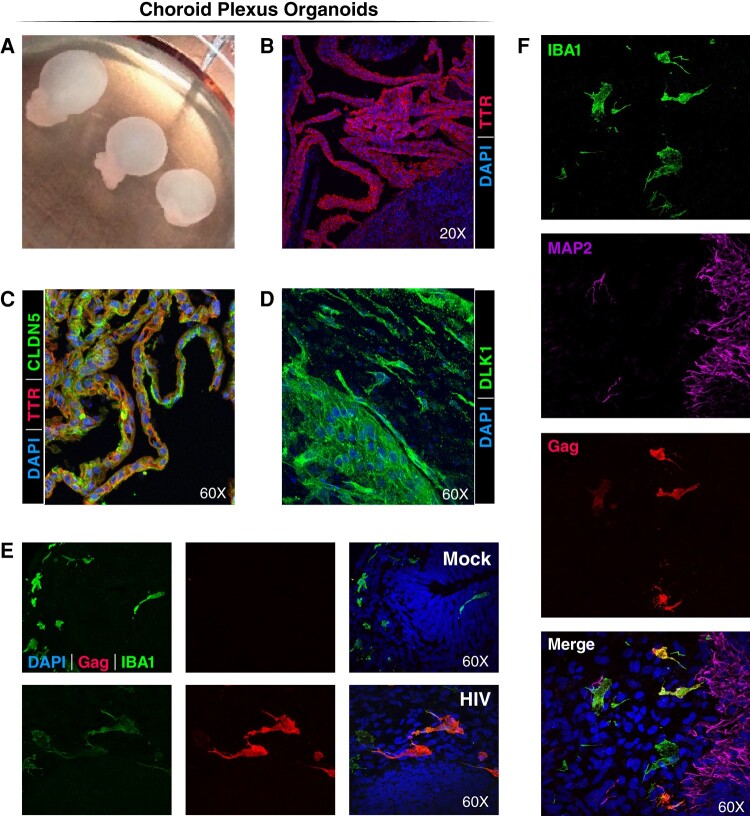
HIV infects microglia in ChP organoids. A) Representative images of ChP organoids at day 58 from one of three experiments. Note fluid-filled “balloon-like” structures corresponding to a form fruste ventricle filled with cerebral spinal fluid (CSF) produced by the choroid plexus cells. B) Immunofluorescence confocal images of ChP epithelia cells stained with an antibody specific TTR (a choroid plexus marker) and DAPI. C) Immunofluorescent confocal images of ChP epithelia cells stained with anti-TTR antibodies and antibodies reacting with claudin 5 (CLDN5), a tight junction marker and with DAPI. D) Immunofluorescence confocal images of ChP stroma cells stained with an antibody specific for DLK1, a marker expressed in the marginal and pseudostratified cells of the third ventricle in the fetal brain. E) ChP organoids were infected or mock infected at day 17 for 4 days with R5-tropic HIV-1 ADA and analyzed at day 50. Immunofluorescent confocal images of microglia stained with antibodies specific for IBA1 or HIV-1 Gag, and DAPI. A subset of the IBA1+ microglia appear HIV Gag positive supporting effective infection of these cells. Magnification is 60× for all panels. F) ChP organoids were infected with HIV-1 ADA on day 17 and analyzed on day 57. Immunofluorescence staining detecting HIV (anti-Gag immunostaining), neuronal cells (anti-MAP2 immunostaining), and microglia cells (anti-IBA1 immunostaining). Magnification is 60× for all panels.

Cerebrospinal fluid (CSF) is produced by the choroid plexus (ChP) present in each of the ventricles of the brain. The choroid plexus contains a capillary rich core surrounded by a single layer of cuboidal epithelial cells whose tight junctions form the blood-cerebrospinal fluid barrier. This barrier regulates the movement of molecules between the systemic circulation and CSF (33). Cells like macrophages, microglia, dendritic cells, and T cells also traffic through ChP to gain entry into the CSF. Similarly, HIV may gain entry into the CNS through the choroid plexus. Using a method described by Ormel et al. (34), we generated ChP organoids containing microglia using two iPSC lines (WT-iPSC and MG-iPSC). After 7–8 weeks, ChP brain organoids containing chambers filled with cerebrospinal fluid were evident (Fig. 2A). Transthyretin (TTR) corresponds to a choroid plexus marker. This protein mediates transport of thyroid hormones and retinol from the bloodstream into the cerebrospinal fluid. The ChP organoids displayed columnar and cuboidal epithelia expressing TTR as well as the presence of tight junctions as indicated by claudin 5 staining. These ChP stromal cells also expressed Delta Like noncanonical notch ligand 1 (DLK1) commonly detected in the marginal and pseudostratified cells of the third ventricle of fetal brain (35) (Fig. 2B–D). We conclude that the established brain organoids generated in vitro correspond to ChP tissue.

To understand the potential interplay of HIV with ChP organoids, we initiated HIV infection at day 17 and cultured the cells for an additional 30 days before immunostaining. As in the cerebral organoids, microglia were principal targets of HIV infection in the ChP organoids as confirmed by dual immunostaining with antibodies specific for IBA1 and Gag (Fig. 2E). In accordance with our earlier studies, direct HIV infection of neurons was not observed (Fig. 2F).

### ART markedly reduces HIV replication but does not completely eliminate inflammation in ChP organoids

**Fig. 3.**
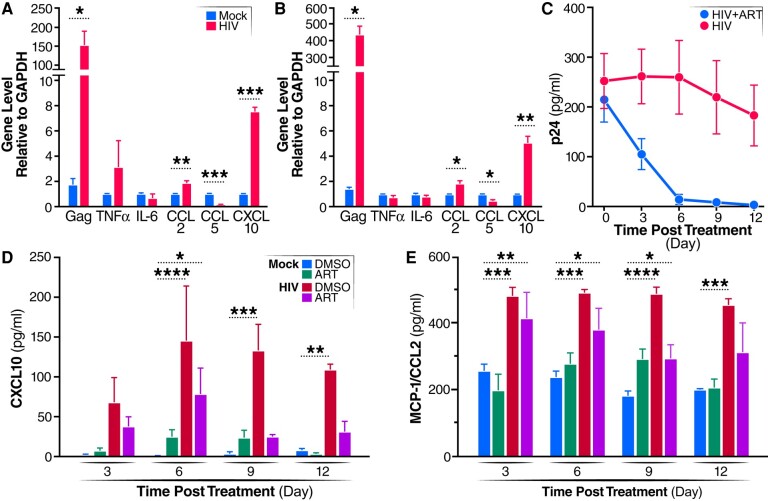
HIV infection of microglia induces inflammation in ChP organoids only partially suppressed by ART. A, B) ChP organoids were infected with R5-tropic HIV-1 ADA and expression of the indicated genes was quantified by RT-qPCR at 30 days (A) or 60 days (B) post-infection. All data represent means ± SEM (*n* = 4 organoids) obtained in three independent experiments. C) ChP organoids were infected with 10 ng of HIV-1 ADA or mock-treated for 50 days, and then exposed to an ART cocktail (doravirine, darunavir, and enfuvirtide, 20 nM final concentration for each antiviral). Aliquots of supernatants were collected at the indicated time points. Viral titers in supernatant samples were determined by p24 ELISA. Data presented represent means ± SD (*n* = 4 organoids) obtained in three independent experiments. D, E) Expression of CXCL10 (D) and CCL2 (E) as determined by ELISA. All data represent means ± SD (*n* = 4 organoids) derived from three independent experiments. *P < 0.05; **P < 0.01; ***P < 0 0.001.

To analyze whether HIV infection alters the expression of various cytokines and chemokines in the ChP organoids, RT-qPCR employing specific cytokine/chemokine primers was used to study the infected ChP organoids. CCL2 and CXCL10 transcripts were upregulated after 30 days (Fig. 3A) and 60 days (Fig. 3B) of infection, as were IFN-a, IFN-b, ISG15, and IFI16 transcripts (Fig. S3A and B). Notably, transcription of CCL5, a natural ligand of CCR5 and a potent HIV-1 entry inhibitor (36), was reduced during HIV infection at both of 30 and 60 dpi.

To test whether ART effectively inhibits HIV infection in the ChP organoids, enfuvirtide, darunavir, and doravirine (all at 20 nM final concentration) were added after 50 days of HIV infection, and p24-Gag protein levels were monitored in the supernatant by ELISA every 3 days. ART significantly reduced HIV replication in the ChP organoids (Fig. 3C). ART also attenuated but did not eliminate the increase in CCL2 and CXCL10 expression seen in supernatants obtained 3, 6, 9, and 12 dpi compared to the mock-infected control group (Fig. 3D, E). These findings suggest that the inflammatory response occurring in response to HIV infection is only partially inhibited by the addition of ART. In the ChP, CCL2 and CXL10 may serve to recruit more immune cells into the brain, including T cells and monocytes that contribute to the ongoing low levels of inflammation that could contribute to development of mild forms of HAND.

### HIV infection activates an inflammatory response in microglia present in ChP organoids

**Fig. 4.**
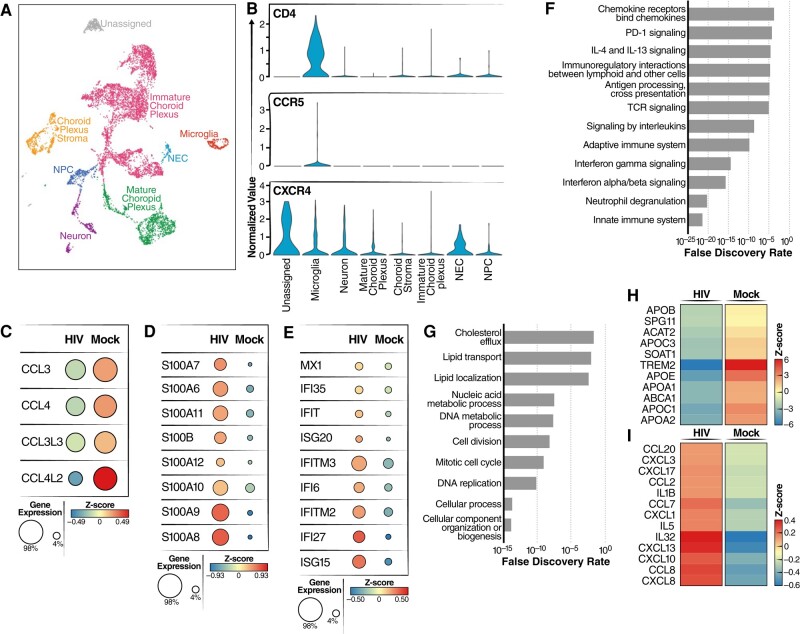
HIV infection induces an inflammatory response in microglia. A) UMAP plot of integrated dataset containing 8,000 single cells from all conditions depicting the presence of various labeled cell types. In the ChP organoids, essentially no astrocytes were identified. Cell types were identified including microglia, neuron, mature choroid plexus, choroid stroma cells, neural progenitor cells (NPC), neuroepithelial cells (NEC), and immature choroid plexus. B) Violin plots of scRNA-seq analyses showing RNA expression levels for different HIV receptors/coreceptors (CD4, CCR5, CXCR4). Note microglia was the only cell type where CCR5 expression was readily detected. C) Dot plots showing expression levels of CCL3, CCL4, CCL3L3, and CCL4L2 in microglia from HIV-infected vs mock-infected ChP organoids. Circle color indicates quantity of chemokine produced and circle size indicates the fraction of cells expressing the specific chemokine. D) Dot plots showing expression levels of S100 calcium-binding proteins in microglia from HIV-infected vs mock-infected ChP organoids. E) Dot plots showing expression levels of type I interferon stimulated genes in microglia from HIV-infected vs mock-infected ChP organoids. F, G) Pathway analysis was performed using an Enrich online tool and the reactome database. The enrichment analysis was performed for differentially upregulated (F) or downregulated (G) genes present in microglia from HIV-infected vs mock-infected ChP organoids. H, I) Heatmaps showing expression levels of lipid transport genes (H) and inflammatory genes (I) in microglia from HIV-infected vs mock-infected ChP organoids.

To determine how HIV infection impacts individual host cells, we performed single-cell RNA-seq (scRNA-seq) on HIV-infected ChP organoids. Distinct cell types within the organoids, including neural progenitor cells, neurons, microglia, mature, and immature choroid plexus cells, stroma, and neural epithelia cells were identified based on cell-specific marker gene expression patterns (Fig. 4A). Next, we examined expression of HIV receptor genes, including CD4, CXCR4, and CCR5 within these populations. CCR5 was only expressed in microglia cells, and at low levels. CD4 was mainly expressed in microglia while CXCR4 was detected in in a much broader array of cell types (Fig. 4B). These results are consistent with findings recently reported by Gumb et al. (37).

To examine changes in gene expression occurring in microglia infected with HIV, we enriched these cells using CD11B+ microbeads. scRNA-seq analyses revealed HIV infection downregulated expression of CCL3L3, CCL3, CCL4L2 and CCL4 (Fig. 4C). These chemokines have been reported to exert anti-HIV activity (38, 39) and thus their downregulation could encourage further viral spread. In contrast, genes involved in innate immune responses, neutrophil degranulation, and inflammatory responses were upregulated (Fig. 4F). Numerous interferon-responsive genes were also activated, including MX1, ISG15, ISG20, IFI27, and IFITM3. Similarly, HIV infection upregulated the S100 family of genes, including S100B, S100A6, S100A7, S100A8, S100A9, S100A10, S100A11, and S100A12 (Fig. 4D and E). Inflammation is induced by S100 family members by binding to specific receptors including the toll-like receptor 4, CD147/EMMPRIN (extracellular matrix metalloproteinase inducer), Receptors for advanced glycation, and specific G-protein-coupled receptors. These binding events lead to subsequent activation of the NF-kappa B and AP1 transcription factor pathways promoting an inflammatory response (40). Of note, increased expression of S100B, S100A4, S100A6, S100A8, and S100A9 has also been linked with Alzheimer's disease (41). RT-qPCR validated increased expression of RNAs corresponding to S100 family members (Fig. S3D), and immunostaining further confirmed increased S100A9 protein in microglia (Fig. S3C). Additionally, several other chemokine/cytokine genes involved in inflammatory responses were upregulated including CCL2, CCL8, CXCL13, CXCL10, and IL32 (Fig. 4I).

In contrast, various microglial genes were specifically downregulated following HIV infection including the TREM2 receptor and ABCA1 transporter (Fig. 4G and H). In brain, TREM2 is predominantly expressed on microglia, and loss of TREM2 reduces phagocytosis and anti-inflammatory activity within these cells (42). Reduction in ABCA1 transporter expression impairs cholesterol efflux, a response linked to increased inflammation (43). These results suggest that the upregulation of the interferon-related and S100 family of genes coupled with downregulation of ABCA1/TREM2 may combine to induce inflammation in HIV-1 infected brain tissue.

**Fig. 5.**
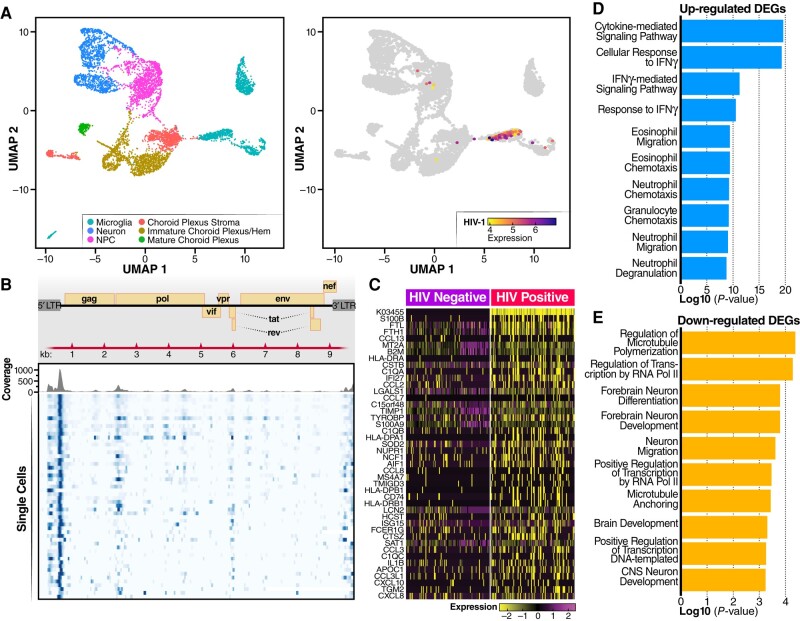
HIV-infected microglia express specific HIV and host genes. A) HIV transcripts are predominantly detected in microglia with minor signals occurring in choroid plexus stroma and neuroprogenitor cells. B) Alignment track of HIV transcripts in all single cells of microglia from HIV infection group across the annotated HIV AD8 consensus sequence. Top panel: read coverage displayed as histogram; bottom panel: pile-up view of the individual reads. C) Heatmap showing highly expressed genes in HIV-positive microglia compared with uninfected microglia. D, E) GO enrichment showing the biological processes corresponding to significantly upregulated and downregulated genes in HIV-infected microglia.

To examine the gene expression patterns in HIV-infected rather than bystander uninfected microglia, we realigned all the single-cell sequencing reads with the HIV-1 AD8 genome sequence to identify HIV-infected RNA+ cells. As expected, no HIV transcripts were detected in noninfected organoids, but viral RNA sequences were detected in the infected organoids primarily in microglia (Figs. 5A and B, S3E). As anticipated based on the 3′10x Genomics single-cell assay and mapping to a consensus reference sequence, alignment of HIV transcripts in microglia was biased to the 3′ and 5′ long terminal repeat regions (Fig. 5B). Compared to bystander uninfected microglia, the infected microglia exhibited upregulation of S100 family genes (S100B and S100A9), several chemokines including CCL13, CCL2, CCL8, CXCL10, and CXCL8, and genes indicating microglia activation, AIF1 and CD74 (Fig. 5C). Pathway analysis revealed upregulation of genes involved in cytokine-mediated and interferon-gamma-mediated signaling, as well as neutrophil degranulation (Fig. 5D). GO analyses of downregulated genes in infected microglia revealed an enrichment for genes involved in regulation of microtubule polymerization/depolymerization, RNA polymerase II transcription, and genes involved in neuron migration and forebrain neuron differentiation (Fig. 5E). Together, these results highlight how HIV infection of microglia triggers an inflammatory response leading to microglia dysfunction.

### HIV infection elicits a broader inflammatory response involving uninfected stromal cells, mature ChPs, immature ChPs, and NPC

**Fig. 6.**
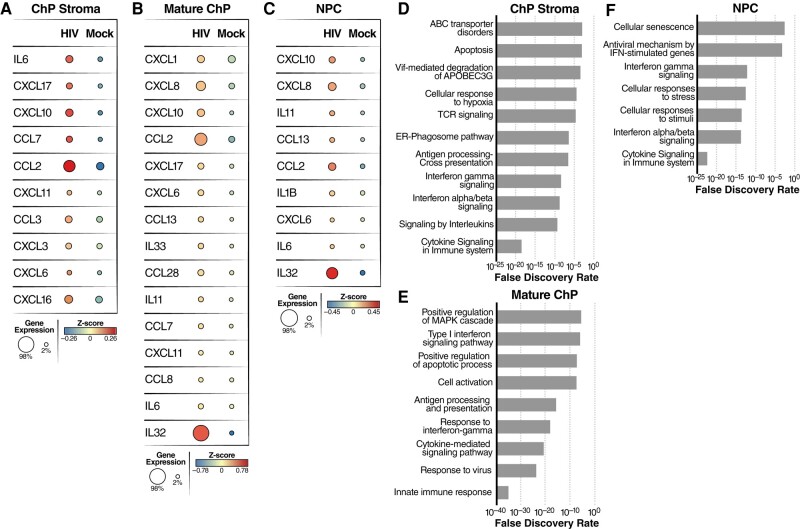
HIV infection of microglia leads to upregulation of inflammatory gene expression in multiple cell types including ChP stroma, mature ChP and NPC. A–C) Dot plots derived from scRNA-seq data depicting both the average level of expression (color) and number of expressing cells (dot size) for specific inflammation-related genes identified in ChP stroma (A), mature ChP (B), and NPC (C). D–F) Pathway analysis was performed using the Enrich online tool and the reactome database. The enrichment analysis was performed for differentially upregulated genes in ChP stroma (D), mature ChP (E), and NPC (F) from HIV-infected vs mock-infected ChP organoids.

**Fig. 7.**
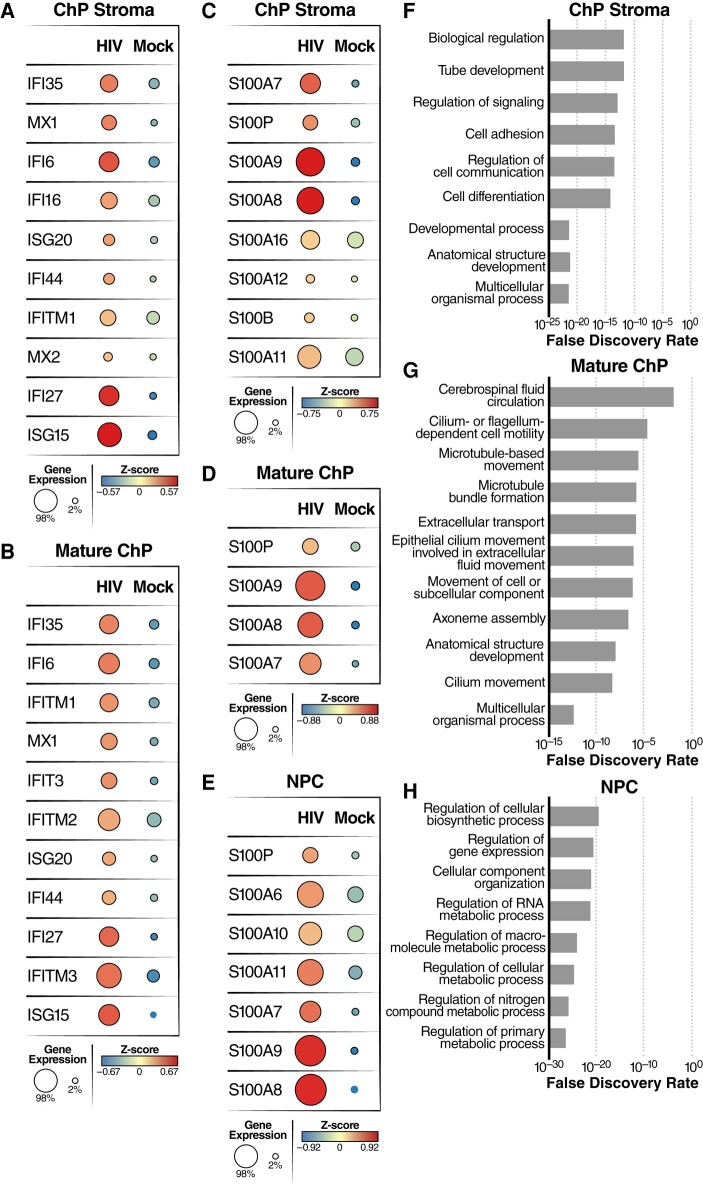
HIV infection of microglia leads to upregulation of inflammatory gene expression in multiple cell types including ChP stroma, mature ChP and NPC. A, B) Dot plots showing average expression and number of cells expressing the interferon-responsive genes in ChP stroma (A) and mature ChP (B). C–E) Dot plots showing average expression and number of cells expressing the S100 family of genes in ChP stroma (C), mature ChP (D), and NPC (E). F–H) Pathway analysis was performed using Enrich online tool and the GO database. The enrichment analysis was applied to ChP stroma (F), mature ChP (G), and NPC (H) for HIV-infected vs mock-infected organoids.

To determine how HIV infection impacts the expression of host genes in cell types other than microglia, we analyzed mature ChP, ChP stromal cells, neural progenitor cells (NPC), and immature ChP cells from our scRNA-seq datasets. Following infection, we observed upregulation of many inflammatory cytokines/chemokines including IL6, CCL2, CCL3, CCL7, CXCL3, CXCL6, CXCL10, CXCL11, CXCL16, and CXCL17 in ChP stroma cells (Fig. 6A); CXCL1, CXCL8, CXCL10, CCL2, CXCL17, IL6, and IL32 in mature ChP (Fig. 6B); CXCL10, CXCL8, CCL2, and IL32 in NPCs (Fig. 6C); and CXCL17, CXCL10, CXCL16, IL11, CCL2, and IL32 in immature ChP (Fig. S4B). Pathway analysis revealed that innate immune response, cytokine signaling in the immune system, and apoptosis were occurring in these cell types (Figs. 6D–F and S4A). Closer inspection of the upregulated genes in ChP stroma cells and mature ChP cells also identified several interferon-inducible genes including ISG15, IFI35, IFI6, and MX1 (Fig. 7A and B). After HIV infection, S100 family genes, including S100A7, S100A8, S100A9, and S100P, were significantly upregulated in ChP stroma, mature ChPs, NPCs, and immature ChPs (Figs. 7C–E and S4C). Several genes involved in cellular responses to HIV infection, including LCN2, ICAM1, SOD2, TNIP1, and INHBA, were also significantly upregulated during HIV infection (Figs. S4D and S5) (44–48). Notably, LCN2 and SOD2 have been strongly linked to HIV-associated neuronal damage (46, 47).

Genes involved in multicellular organismal processes, cell differentiation, regulation of cell communication, and biological regulation, were downregulated in ChP stroma following HIV infection (Fig. 7F). GO analysis of HIV-downregulated genes in mature ChPs revealed an enrichment of genes associated with cilium-mediated motility, axoneme assembly, epithelial cilium movement including extracellular fluid movement, cilium or flagellum-dependent cell motility, and CSF circulation (Fig. 7G). Taken together, these findings suggest that HIV infection disrupts cellular processes involved in cell movement and fluid circulation. In addition, multiple metabolic processes linked with RNA, nitrogen compounds, and macromolecules were downregulated in NPCs (Fig. 7H). Overall, our findings reveal that HIV infection of microglia induces robust changes in the gene expression profiles of bystander cells suggesting the inflammatory response occurring in microglia fundamentally alters the cellular microenvironment in a way that favors the broader spread of inflammation.

### HIV infection increases neuroinflammation, reduces neuronal metabolism, and impairs neurotransmitter transport

**Fig. 8.**
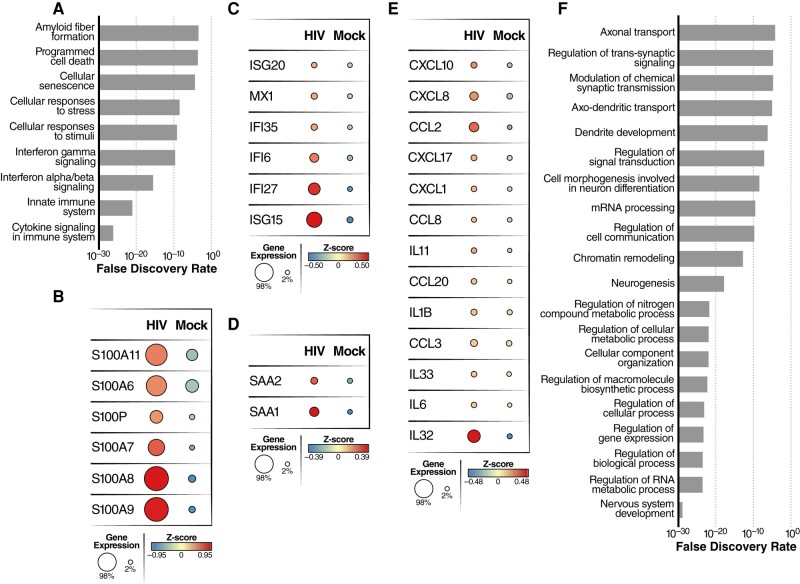
HIV infection of microglia induces inflammation and alters expression of genes required for normal neuronal function. A) Pathway analysis showing differentially upregulated genes in neurons from HIV-infected vs mock-infected ChP organoids. B–E) Dot plots showing average expression and number of cells expressing S100 family genes (B), interferon-related genes (C), serum amyloid A (D), and inflammation-related genes (E) in neurons from HIV-infected vs mock-infected ChP organoids. F) Pathway analysis was performed using the Enrich online tool using the GO database. The enrichment analysis was performed for differentially downregulated genes in neurons from HIV-infected vs mock-infected ChP organoids.

Since neuronal dysfunction and death play central role in HAND, we next focused attention on changes in neuronal gene expression that occurred following HIV infection of microglia in the organoids. Analysis of the scRNA-seq data for neurons revealed increased expression of genes involved in the innate immune response, cytokine signaling in the immune system, cellular senescence, and cell death (CASP1, PMAIP1, BID, and PERP) (Fig. 8A); genes related to inflammation or neurodegenerative disease, such as S100A6/A7/A8/A9 and S100P (Fig. 8B); and genes associated with type I interferon production including ISG15, ISG20, IFI27, IFI35, IFI6, and MX1 (Fig. 8C). Genes encoding the serum amyloid A proteins SAA1 and SAA2, which are involved in amyloid plaque aggregation, were also upregulated in neurons present in infected ChP organoids, suggesting inflammation-induced neuronal damage/degeneration (49, 50) (Fig. 8D). This finding was further corroborated by increased expression of a number of proinflammatory chemokine/cytokine genes (CCL2, CCL8, CXCL10, CXCL8, IL1B, IL6, and IL32) (Fig. 8E). Intriguingly, HIV infection also increased neuronal expression of the HIV Nef-interacting genes ARF1 and AP1S1 (Fig. S5F) raising the possibility that extracellular Nef might play an unidentified role in neurotoxicity. We also observed that LCN2 and SOD2, two biomarkers of HAND (46, 47), were markedly elevated in neurons following HIV infection of the organoids (Fig. S5B).

GO analysis of downregulated genes in neurons revealed enrichment for genes involved in nervous system development, generation of neurons, neurogenesis, RNA/nitrogen compound metabolic process, macromolecule biosynthetic process, cell communication, axo-dendritic transport, modulation of chemical synaptic transmission, and regulation of trans-synaptic signaling (Fig. 8F). Together, these findings underscore how HIV infection of microglia profoundly alters the gene expression profile of bystander neurons likely driving major changes in neuronal function, synaptic transmission, and viability.

## Discussion

While ART reduces the severity of HIV-associated brain disease, up to 30–50% of HIV-positive individuals continue to exhibit milder forms of HAND. The pathophysiological changes associated with HAND are not well understood due to a paucity of informative experimental models and limited access to useful HIV-infected human brain samples. Here, we describe two brain organoid models and their use to examine the effects of HIV infection on multiple brain cell types. We chose brain organoids for these studies because they have proven valuable in investigation of various neurodevelopmental abnormalities including autism spectrum disorders and microcephaly associated with Zika virus infection (51–54). However, most of these prior studies utilized brain organoids lacking microglia, the primary cellular target for HIV in the brain. To address this shortcoming, we differentiated engineered iPSC cells into microglia and incorporated these cells into developing iPSC-derived brain organoids. In studies of both cerebral and ChP organoids, we find that HIV predominantly infects microglia in agreement with prior studies (8, 55–57). Furthermore, our findings suggest that astrocytes do not support productive HIV replication and are likely poorly infectable due to limited CCR5 expression.

A likely cause of HAND symptoms is chronic immune activation and inflammation, which persists in people living with HIV at low levels despite addition of ART (58, 59). Indeed, although ART effectively suppresses HIV replication in ChP organoids, it does not completely abrogate production of the proinflammatory chemokines (CXCL10 and CCL2). In the pre-ART era, CXCL10 and CCL2 were regarded as biomarkers for HAND. CCL2 and CXCL10 expression are also increased in the CSF of people living with HIV (60) as well as in SIV-infected models of simian encephalitis (61). Our experiments show that one of the cellular sources of CXCL10 and CCL2 is infected microglia. In addition, these infected microglia upregulate numerous type I interferon-responsive genes (ISG15, ISG20, MX1, and IFI6), as well as S100 calcium-binding proteins (S100B, S100A6/A7/A8/A9) (62, 63). The S100 family of proteins are known mediators of inflammation but also help regulate a variety of other intracellular and extracellular responses including cell death, proliferation, differentiation, and migration (40). In addition to their role in inflammation, recent studies indicate that the S100B, S100A6, S100A8, and S100A9 proteins play a role in aging and neurodegeneration (40, 64–67). Numerous members of the S100 family are increased in the brains of Alzheimer's disease patients as a result of astrocyte and microglial activation (41). Not only does S100A8/A9 operate as an inflammatory response gene, it also amplifies inflammatory signals by promoting the release of proinflammatory cytokines and activating NF-κB, a “master regulator” of inflammation (68). These findings suggest that the upregulation of S100 family proteins could serve as an additional genetic biomarker for HAND. Another contributor to brain inflammation could be the downregulation of the ABCA1 transporter and TREM2 receptor that occurs in microglia following HIV infection. ABCA1 transports cholesterol out of cells for assembly into high-density lipoproteins (69–71). ABCA1 and APOE deficiency promotes inflammation in macrophages (43, 72), suggesting that intracellular accumulation of cholesterol likely triggers an inflammatory response. TREM2 is essential for maintaining microglial metabolic fitness. TREM2 deficiency enhances inflammation and impairs phagocytosis (42).

However, while microglia appear to form the principal target for HIV infection, other organoid cells beyond microglia develop signatures of inflammation following HIV infection. Increased expression of CCL2 and CXCL10 and several S100 family members (notably S100 A8/A9) is observed in neurons, mature ChP cells, in infected ChP organoids. Self-sustaining and amplifying cycles of inflammation extending beyond the infected cells may also help explain why ART is not entirely effective in preventing HAND. Of note, IL32, a proinflammatory cytokine that induces IL-6, IL-1β, and various chemokines is significantly elevated across multiple cell types during HIV infection. IL32 is a robust biomarker of control failure in HIV-infected slow progressors, and of persistent inflammation in virologically suppressed HIV-infected individuals (73, 74). Our study also identifies other elevated genes including SAA1/2, ICAM1, SOD2, and LCN2 that are clearly linked to brain injury (46, 47, 50, 75, 76). In this amplifying inflammatory microenvironment, bystander neurons are likely unable to prevent dysfunctional intracellular events that ultimately culminates with neuronal apoptosis.

**Fig. 9.**
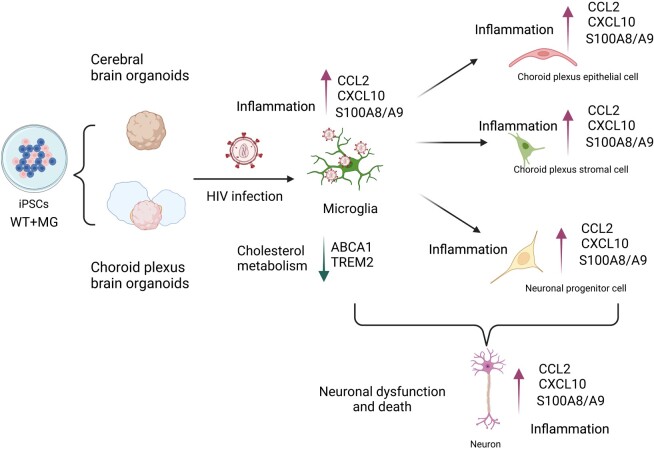
Schematic summary: HIV infects microglia from both iPSC-differentiated cerebral brain organoids and ChP organoids inducing an inflammatory microenvironment leading to neuronal dysfunction. HIV infection of microglia induces an inflammatory response including an increase in S100A8/A9, CCL2, and CXCL10, and disrupts cholesterol homeostasis, resulting in microglia dysfunction. HIV infection of microglia induces robust changes in IFN stimulated genes, inflammatory genes and S100 family genes in uninfected bystander cells including stromal cells, mature ChPs, immature ChPs, and NPC. The inflammation and released HIV toxic proteins driven by HIV infection of microglia also induces neuroinflammation in bystander neurons and appears to impair neuronal function by disrupting synaptic transmission and axo-dendritic trafficking. These events likely play a key role in the progressive development of HAND.

In summary, we propose that infected microglia are responsible for triggering an inflamed and toxic microenvironment that ultimately leads to neuronal cell dysfunction and death (Fig. 9). According to this model, viral infection principally occurs within microglia and initiates an inflammatory response that is propagated to other cells within the organoid including mature and immature ChP cells, as well as the surrounding stromal cells and NPCs. This inflammatory response centrally involves HIV-associated increases in the expression of S100 calcium-binding proteins, IFN, and various cytokines/chemokines coupled with downregulation of genes involved in cholesterol efflux (ABCA1and TREM2). All these events promote and amplify the inflammatory response allowing spread to bystander cells importantly including neurons. Derangements in neuronal metabolism and decreased neurotransmitter transport combine to promote neuronal cell dysfunction and death. We believe these organoid models represent a highly useful experimental platform to explore in even greater depth the pathogenetic mechanisms underlying HAND development. Our findings suggest that the use of anti-inflammatory drugs could be a promising option for treating individuals with HAND. Additionally, this platform could be employed to assess other strategies beyond the use of anti-inflammatory drugs, to mitigate the low levels of neuroinflammation that likely drive the mild cases of HAND observed in some individuals on long-term ART.

## Materials and methods

### Viruses and cells

HIV ADA (NIH AIDS Reagent Program) was used for infection of the human brain organoid. Infections were performed in a biosafety level 3 (BSL3) laboratory. Viral titers were determined by p24 ELISA.

### iPSC maintenance

Many of the Materials and Methods involving preparation of the brain organoids and their analysis have been described in our prior publication and are repeated in part or whole here (77). Wild-type WTC11 iPSC and microglia-iPSC (32) (MG-iPSC) were cultured in mTeSR plus (Stemcell Technologies) on Matrigel (8 μg/mL, Corning)-coated cell culture plates at 37°C, 5% CO_2_. Cells were passaged every 4–5 days using ReLeSR (Stemcell Technologies) and treated with Rock Inhibitor Y-27632 (SelleckChem) for 24 h after each passage.

### Brain organoid differentiation

Cerebral organoids were formed with the commercial STEMdiff™ cerebral organoid kit following manufacturer's instructions (Stemcell Technologies) (77). Single-cell suspensions of human iPSC colonies were prepared by addition of a gentle cell dissociation reagent (Stemcell Technologies). Around 9,000 cells (2,500 MG + 7,500 WTC11) were seeded into each well of an ultra-low attachment V-bottom 96 well plate to allow embryoid bodies to form in the presence of 20 uM Y27632. Subsequently, 100 μL of EB medium containing 2 μg/mL of doxycycline was added to each well on days 2 and 4 without disturbing the EBs. EBs were cultured for an additional 3 days after being relocated to 24-well low-attachment plates containing neural induction medium on day 5. The EBs were then suspended in 15 μL of Matrigel and cultured in neural expansion medium for an additional 3 days in 6-well low-attachment plates to allow organoid formation to continue. Finally, the organoids were cultured in neural culture medium and placed on an orbital shaker for further development.

Choroid plexus organoids were generated according to a previously published protocol briefly described below (34). In total, approximately 8,000 cells (4,000 MG + 4,000 WTC) were seeded into each well of a V-bottom ultra-low-attachment 96-well plate in EB formation media (DMEM/F12, 1% Glutamax supplement (vol/vol), 1% MEM-NEAA, 3% ES-quality FBS, 20% KOSR, 0.1 mM 2-mercaptoethanol, and 4 ng/mL bFGF) supplemented with 20 μM Y27632. Without disrupting the EBs, 100  μL EB medium containing 2 µg/mL of doxycycline were added to each well on day 2 and day 4. On day 6, EBs were transferred to 24-well low-attachment plates in neural induction medium (DMEM/F-12, 1 mM GlutaMAX, 0.1 mM MEM-NEAA, 1% penicillin/streptomycin, 1% N2 supplement [Gibco], and 0.1 μg/mL heparin) for another 6 days. EBs were further embedded in 15 μL of Matrigel and cultured in neural expansion medium (1:1 ratio of neurobasal medium [Gibco] and DMEM/F-12, 1% B27 supplement without vitamin A [Gibco], 1% penicillin/streptomycin, 0.5% N2 supplement, 0.05 mM MEM-NEAA, and 0.05 mM 2-mercaptoethanol and 2.5 μg/mL insulin) in 6-well low-attachment plates for 4 days to permit organoid formation. Neural culture medium (1:1 ratio of DMEM/F-12 and neurobasal medium (Gibco), 1% B27 supplement with vitamin A [Gibco], 1% penicillin/streptomycin, 0.5% N2 supplement, 0.05 mM MEM-NEAA, 0.05 mM 2-mercaptoethanol, and 2.5 μg/mL insulin) was subsequently used for growing the organoids on an orbital shaker.

### Immunocytochemistry

Brain organoids were fixed overnight at 4°C in 4% paraformaldehyde, followed by three washes with phosphate-buffered saline (PBS) (77). Post-fixation, the organoids were dehydrated in 30% sucrose-PBS solution at 4°C. Subsequently, the organoids were embedded in optical cutting temperature (OCT) compound (VWR) and frozen using dry ice. The frozen tissue was sectioned into 10  μm slices using a cryostat and placed on ultra-frosted glass microscope slides. These sections were stored at −80°C until further use. For permeabilization, the sections were treated with 0.25% Triton X-100 and then blocked using a PBS containing 5% normal goat serum and 2% BSA for 2 h at room temperature. Sections were incubated overnight at 4°C with primary antibodies diluted in blocking buffer. The primary antibodies used in this study and their respective dilutions were: MAP2 (1:1000), TUJ1 (1:1000), NESTIN (1:1000), IBA1 (1:1000), GFAP (1:1000), and HIV core antibody (1:100). After washing three times with PBS, the samples were incubated with fluorescently labeled secondary antibodies (Alexa Fluor 647, 568, and 488 conjugates, Invitrogen, diluted 1:500) for 1 h at room temperature. Following another three washes with PBS, glass coverslips were mounted on the samples. Fluorescence signals were captured using an Olympus FV3000 confocal microscope and analyzed using ImageJ software.

### RT-qPCR

Brain organoids were lysed using Qiagen buffer RLT (QIAGEN) containing 1% β-mercaptoethanol (Bio-Rad) (77). Total RNA was extracted using the RNeasy Micro Kit plus (QIAGEN). After elution, concentration and purity of the RNAs were assessed using a NanoDrop 2000c spectrophotometer (Thermo Fisher Scientific). RNA samples (0.2 to 1 μg) were reverse transcribed with the iScript™ cDNA synthesis kit from Bio-Rad. Real-time PCR was conducted using iTaq™ universal SYBR^®^ Green Supermix (Bio-Rad) together with gene-specific primers (see Table S1). The PCR reactions were carried out using a Bio-Rad CFX connect qPCR system under the following conditions: initial denaturation at 95°C for 10 min, followed by 40 cycles of 95°C for 15 s and 60°C for 1 min. The relative gene expression levels were normalized to the GAPDH control and were calculated following the formula: 2 ^(ΔCT of gene−ΔCT of GAPDH).^

### Organoid dissociation for single-cell RNA-sequencing

In brief, 8–10 organoids per condition (mock and HIV infection) were washed twice with ion-free DPBS. A single-cell suspension was prepared using the neural dissociation kit (Miltenyi Biotec) and the gentle MACS™ dissociator following the manufacturer's protocol. Microglia were isolated using CD11b magnetic microbeads (Miltenyi Biotec). The cells were then resuspended in 0.04% BSA in PBS. Cell viability and cell numbers were assessed before being subjected to the 10× RNA-sequencing protocol.

### Single-cell RNA-sequencing

For each condition, we loaded two single-cell suspensions onto a Chromium Next GEM Chip G, targeting an output of 5,000 to 10,000 cells per channel. This chip was then placed in a Chromium Controller v3.1 (10× Genomics) to generate single-cell GEMs and barcodes. Using the Single-cell 3′ Gel Bead and Library v3.1 kit (10× Genomics) in a BSL3 lab, we carried out GEM formation, barcoding, cDNA amplification, and library creation across the six channels. Both the amplified cDNA and final libraries underwent quality assessment on a Bioanalyzer before being sequenced on an Illumina NovaSeq 6000 platform. Any cells exhibiting more than 30% mitochondrial reads were excluded from further analysis. Data analysis was performed using BBrowser 3, and the raw datasets are accessible at the Gene Expression Omnibus under the accession code GSE262349.

### Statistical analyses

All data are presented as mean ± SEM. Statistical analyses for the qPCR and ELISA experiments were conducted using GraphPad Prism 9 software, incorporating one-way ANOVA.

## Supplementary Material

pgae179_Supplementary_Data

## Data Availability

All data are included in the manuscript and/or supporting information. Materials will be shared upon request.
