# Supplementary material for: Neuroinflammation generated by HIV-infected microglia promotes dysfunction and death of neurons in human brain organoids
Source: PNAS Nexus. 2024 Apr 29;3(5):pgae179. doi: 10.1093/pnasnexus/pgae179 (PMC11086946; doi:10.1093/pnasnexus/pgae179)
Supplement: pgae179_Supplementary_Data [file pgae179_supplementary_data.docx]

**
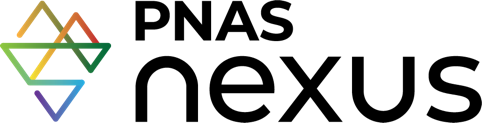
**

**Supplementary Information for**

**Neuroinflammation generated by HIV-infected microglia promotes dysfunction and death of neurons in human brain organoids**

Weili Kong^1,2^, Julie Frouard^1,2,5^, Guorui Xie ^1,2,5^, Michael J. Corley^6,7^, Ekram Helmy^1,2^, Gang Zhang^1,2^, Roland Schwarzer ^1,2^, Mauricio Montano^1,2^, Peter Sohn^3^, Nadia R. Roan^1,2,5,^, Lishomwa C. Ndhlovu^6,7^,

Li Gan^7,8^ and Warner C. Greene^1,2,4*^

^1^Michael Hulton Center for HIV Cure Research at Gladstone, San Francisco, CA, USA 94158

^2^Gladstone Institute of Virology, San Francisco, CA, USA 94158

^3^Gladstone Institute of Neurological Disease, San Francisco, CA, USA 94158

^4^Departments of Medicine and Microbiology and Immunology, University of California, San Francisco, San Francisco, CA, USA 94143

^5^Department of Urology, University of California, San Francisco, San Francisco, CA, USA 94143

^6^Division of Infectious Diseases, Department of Medicine, Weill Cornell Medicine, New York, NY, USA 10021

^7^Brain and Mind Research Institute, Weill Cornell Medicine, NY, New York, USA.

^8^Helen and Robert Appel Alzheimer's Disease Research Institute, Weill Cornell Medicine, NY, New York, USA.

^*^Address correspondence to Warner C. Greene, email: [warner.greene@gladstone.ucsf.edu](mailto:warner.greene@gladstone.ucsf.edu)

This document includes

Figures S1 to S5

Tables S1-S2

**Figure S1.**

(A) Representative images of cerebral organoids at week 8.

(B) Representative images of the ventricular zone (VZ)-like structure formed within the cerebral organoids (neuron marker: MAP2, green) and neural progenitor cells (NPC markers: SOX2, purple) in cerebral organoids at week 4.

(C) Confocal images demonstrating the presence of astrocytes (GFAP, red) and neurons (MAP2, green) in the cerebral organoids at week 8.

(D) Confocal images demonstrating the presence of microglia (IBA1, purple) and neurons (TUJ1, green) in the brain organoids at week 8.

**

**

***

**

***

**Figure S2.**

(A) Cerebral organoids were infected with 10 ng of HIV-1 ADA or mock-infected at day 90 for 2 days followed by addition of an ART cocktail (doravirine, darunavir, and enfuvirtide, 20 nM final concentration for each drug). Aliquots of supernatants were collected at the indicated time points. Viral titers present in the culture supernatants samples were determined by p24 ELISA. Error bars indicate mean ± SD (n=3) obtained in three independent experiments. *P<0.05; **P<0.01; ***P<0 .001.

(B) Human cerebral brain organoids were infected with HIV-1 followed by ART treatment. Immunofluorescence confocal images of microglia stained with antibody against IBA1 (red), HIV-1 (green), and with nuclear stain DAPI (blue). Magnification is 60X for all panels.

**Figure S3.**

(A-B) ChP organoids were infected with HIV and expression levels of the indicated genes were quantified by RT-qPCR at 30 days (A) and 60 days (B) post-infection. All data are expressed as means ± SEM (n = 4 organoids) obtained in three independent experiments. *P<0.05; **P<0.01; ***P<0 .001.

(C) Immunofluorescent confocal images of ChP stroma cells stained with antibody specific for S100A9 (green), HIV Gag (red) and with nuclear DAPI stain (blue). Magnification is 20X for all panels.

(D) Human ChP organoids were infected with HIV and expression levels of indicated genes were quantified by RT-qPCR at 60 days post-infection. All data are presented as means ± SEM (n = 4 organoids) obtained in three independent experiments. *P<0.05; **P<0.01; ***P<0 .001.

(E) HIV transcript expression in mock infected versus HIV-infected ChP organoids. *P<0.05; **P<0.01; ***P<0 .001; ****P<0 .0001.

**Figure S4.**

(A) Pathway analysis showing differentially upregulated genes in immature ChP cells in HIV-infected versus mock-infected ChP organoids.

(B) Dot plot showing average expression and number of cells expressing inflammation-related genes in immature ChP organoids.

(C) Dot plot showing average expression and number of cells expressing the S100 family genes in immature ChP cells in HIV-infected versus mock infected ChP organoids .

(D) Dot plot showing average expression and number of cells expressing TNIP1, ICAM1, INHBA, SOD2 and LCN2 genes in immature ChP cells in HIV-infected versus mock infected ChP organoids

**Figure S5.**

(A-C) Dot plots showing average expression and number of cells expressing TNIP1, INHBA, SOD2 and LCN2 genes in microglia (A), neurons (B) and NPCs(C) from HIV-infected versus mock-infected ChP organoids.

| Gene | Primer sequence (5’-3’) Forward | Primer sequence (5’-3’) Reverse |
| --- | --- | --- |
| Gag | GAC GCT CTC GCA CCC ATC TC | CTG AAG CGC GCA CGG CAA |
| TNF-a | CCTGGGATTCAGGAATGTGTG | TGTAGGCCCCAGTGAGTTCTG |
| IL-6 | ATGAGGAGACTTGCCTGGTGA | ATCTGCACAGCTCTGGCTTGT |
| CCL2 | GAGAGGCTGAGACTAACCCAGA | ATCACAGCTTCTTTGGGACACT |
| CCL5 | GCTGTCATCCTCATTGCTACTG | TGGTGTAGAAATACTCCTTGATGTG |
| CXCL10 | TGCCATTCTGATTTGCTGCC | TGCAGGTACAGCGTACAGTT |
| IFN-a | GCTTTACTGATGGTCCTGGTGGTG | GAGATTCTGCTCATTTGTGCCAG |
| GAPDH | GCCTCTTGTCTCTTAGATTTGGTC | TAGCACTCACCATGTAGTTGAGGT |
| IFN-b | GAATGGGAGGCTTGAATACTGCCT | TAGCAAAGATGTTCTGGAGCATCTC |
| S100A8 | AGA CCG AGA CCG AGT GTC CTC | TGC CAC GCC CAT CTT TAT |
| S100A9 | TCA AAG AGC TGG TGC GAA A | CAG CTG CTT GTC TGC ATT TG |
| ISG15 | CTCTGAGCATCCTGGTGAGGAA | AAGGTCAGCCAGAACAGGTCGT |
| IFI6 | TGATGAGCTGGTCTGCGATCCT | GTAGCCCATCAGGGCACCAATA |

(D-E) Dot plot showing average expression and number of cells expressing TNIP1, ICAM1, INHBA, SOD2 and LCN2 genes in HIV-infected versus mock-infected ChP stroma (D) and mature ChP cells (E) in ChP organoids.

(F) Dot plot showing average expression and number of neurons expressing AP1S1 and ARF1 genes in HIV-infected versus mock-infected ChP organoids.

Table S1: Primers are used in RT-qPCR.

Table S2: List of materials used in this study.

| **REAGENT or RESOURCE** | **SOURCE** | **IDENTIFIER** |
| --- | --- | --- |
| Antibodies | | |
| Mouse monoclonal anti- HIV core | Beckman | Cat# IMBULK1B |
| Rabbit monoclonal anti-DLK1 | Abcam | Cat# ab21682 |
| Mouse monoclonal anti-TTR | Abcam | Cat# ab204997 |
| Mouse monoclonal anti-Claudin 5 | Abcam | Cat#ab15106 |
| Rabbit monoclonal anti-S100A8+S100A9 | Abcam | Cat#ab288715 |
| Mouse monoclonal anti-MAP2 | Millipore Sigma | Cat#MAB3418 |
| Rabbit polyclonal anti-NESTIN | Millipore Sigma | Cat#ABD69 |
| Rabbit polyclonal anti-IBA1 | FUJIFILM Wako Pure Chemical Corporation | Cat#019-19741 |
| Mouse monoclonal anti-TUJ1 | BioLegend | Cat#801201 |
| Chicken polyclonal anti- MAP2 | EnCor | Cat#CPCA-MAP2 |
| Chicken polyclonal anti- GFAP | EnCor | Cat#CPCA-GFAP |
| Rabbit polyalcohol anti-GFAP | Novus Biologicals | Cat#NB300-141 |
| Mouse monoclonal anti-CD68 | Santa cruz | Cat#sc-20060 |
| Rabbit polyclonal anti-S100A9 | GeneTex | Cat#GTX129575 |
| Rabbit polyclonal anti- Cleaved Caspase-3 (Asp175) | Cell Signaling Technologies | Cat#9661 |
| AlexaFluor 488, 647 goat anti-rabbit IgG (H+L) | Life Technologies | Cat#A11008, A2134003 |
| AlexaFluor 488, 647 goat anti-chicken IgG (H+L) | Life Technologies | Cat#A32931, A32933 |
| AlexaFluor 488, 568, goat anti-mouse IgG (H+L) | Life Technologies | Cat#A11001, A11031 |
| Vector® TrueVIEW® Autofluorescence Quenching Kit with DAPI | VECTOR laboratories | SP-8500-15 |
| Bacterial and virus strains |  |  |
| HIV ADA | Gladstone Institutes |  |
|  |  |  |
| Chemicals, peptides, and recombinant proteins | | |
| Matrigel | Corning | Cat#354234 |
| Scigen Tissue-Plus™ O.C.T. Compound | FisherScientific | Cat#23-730-571 |
| Rock Inhibitor Y27632 | Selleckchem | Cat#S1049 |
| Gentle Cell Dissociation Reagent | Stem Cell Technologies | Cat#100-0485 |
| Doxycycline | Selleckchem | Cat# S5159 |
| Enfuvirtide acetate salt | MilliporeSigma | Cat# SML0934 |
| Darunavir | MilliporeSigma | Cat# SML0937 |
| Doravirine | MedChemExpress | Cat# HY-16767 |
| Critical commercial assays | | |
| Cerebral Organoid kit | Stem Cell Technologies | Cat#08570, 08571 |
| Chromium Next GEM Chip G Single Cell Kit | 10x Genomics | Cat#1000127 |
| Chromium Next GEM Single Cell 3' GEM, Library & Gel Bead Kit v3.1 | 10x Genomics | Cat#1000128 |
| Single Index Kit T Set A | 10x Genomics | Cat#1000213 |
| QIAshredder | QIAGEN | Cat#79654 |
| iTaq Universal SYBR Green Supermix | Bio-Rad | Cat#1725121 |
| RNeasy Plus Micro Kit | QIAGEN | Cat#74034 |
| RNeasy Mini Kit | QIAGEN | Cat#74104 |
| iScript™ cDNA Synthesis Kit | Bio-Rad | Cat#1708891 |
| HIV p24 ELISA kit | Abcam | Cat#ab218268 |
| Human IP-10 ELISA kit | Abcam | Cat#ab173194 |
| Human CCL2 ELISA kit | Abcam | Cat#ab179886 |
| CD11b (Microglia) MicroBeads, human and mouse | Miltentyi Biotec | Cat# 130-093-636 |
| Neural tissue dissociation kit(P) | Miltentyi Biotec | Cat# 130-092-628 |
| Experimental models: Cell lines | | |
| Human: WTC11 iPSC | Gladstone institutes | N/A |
| Human: Modified WTC11 iPSC | Gladstone institutes | N/A |
| Software and algorithms | | |
| ImageJ | Schneider et al., 2012 | https://imagej.nih.gov/ij/ |
| BBrowser | BioTuring | https://bioturing.com/company/aboutus |
| GraphPad Prism 9.0 | GraphPad | https://www.graphpad.com/ |
| Cell ranger | 10x Genomics | https://support.10xgenomics.com/single-cell-gene-expression/software/overview/welcome |
| Adobe Illustrator | Adobe | <https://www.adobe.com/products/illustrator.html> |
| BioRender | BioRender | https://app.biorender.com/ |
